# Epigallocatechin Gallate Attenuates Gentamicin-Induced Nephrotoxicity by Suppressing Apoptosis and Ferroptosis

**DOI:** 10.3390/molecules27238564

**Published:** 2022-12-05

**Authors:** Lin Yue, Ya-Ru Yang, Wen-Xian Ma, Hong-Yan Wang, Qian-Wen Fan, Yue-Yue Wang, Chao Li, Jing Wang, Zi-Mu Hu, Xue-Fu Wang, Feng-He Li, Ming-Ming Liu, Juan Jin, Chao Shi, Jia-Gen Wen

**Affiliations:** 1 Inflammation and Immune Mediated Diseases Laboratory of Anhui Province, Anhui Institute of Innovative Drugs, School of Pharmacy, Anhui Medical University, Hefei 230032, China; 2 Department of Clinical Pharmacology, Second Hospital of Anhui Medical University, Hefei 230601, China; 3 State Key Laboratory of Tea Plant Biology and Utilization, School of Tea and Food Science and Technology, Anhui Agricultural University, Hefei 230036, China; 4 Department of Cardiac Surgery, The First Affiliated Hospital of Bengbu Medical College, Bengbu 233004, China

**Keywords:** nephrotoxicity, gentamicin, apoptosis, ferroptosis, epigallocatechin-3-gallate, Nrf2, GPX4

## Abstract

Gentamicin (GEN) is a kind of aminoglycoside antibiotic with the adverse effect of nephrotoxicity. Currently, no effective measures against the nephrotoxicity have been approved. In the present study, epigallocatechin gallate (EG), a useful ingredient in green tea, was used to attenuate its nephrotoxicity. EG was shown to largely attenuate the renal damage and the increase of malondialdehyde (MDA) and the decrease of glutathione (GSH) in GEN-injected rats. In NRK-52E cells, GEN increased the cellular ROS in the early treatment phase and ROS remained continuously high from 1.5 H to 24 H. Moreover, EG alleviated the increase of ROS and MDA and the decrease of GSH caused by GEN. Furthermore, EG activated the protein levels of nuclear factor erythroid 2-related factor 2 (Nrf2) and heme oxygenase-1 (HO-1). After the treatment of GEN, the protein level of cleaved-caspase-3, the flow cytometry analysis and the JC-1 staining, the protein levels of glutathione peroxidase 4 (GPX4) and SLC7A11, were greatly changed, indicating the occurrence of both apoptosis and ferroptosis, whereas EG can reduce these changes. However, when Nrf2 was knocked down by siRNA, the above protective effects of EG were weakened. In summary, EG attenuated GEN-induced nephrotoxicity by suppressing apoptosis and ferroptosis.

## 1. Introduction

Nephrotoxicity has the symptoms of proteinuria, cylindruria, azotemia, and renal dysfunction. It can be caused by drugs such as cisplatin and aminoglycoside antibiotics [1,2]. Gentamicin (GEN) is a kind of aminoglycoside antibiotic generally used to eradicate the infection of gram-negative bacteria, particularly for the population of children [3]. However, nephrotoxicity and ototoxicity are the common adverse reactions of aminoglycoside antibiotic, which considerably reduce its clinical application. Extensive research has been conducted to prohibit or attenuate these poisons. However, no effective measures have been approved until now.

At present, numerous studies have investigated the mechanism of GEN toxicity, and they have found that intracellular oxidation/antioxidant imbalance, calcium ion disturbance, calcium-activated protease activation, metabolic inhibition, and specific drug accumulation are closely related to its toxicity [4,5]. As a highly polar drug, it enters protopuria through renal filtration soon after its application, and then enters renal tubular cells from urine through endocytosis of the Megalin–Cubilin complex [4,6]. A large amount of cellular GEN has a destructive impact on complex-I activity, leading to the production of excessive ROS [7]. Moreover, ROS can further assault mitochondria and lead to DNA breakage, lipid peroxidation, and activation of inflammatory signals [8]. Thus, GEN-induced ROS is possibly the key node in the damage of renal tubular epithelial cells in GEN-induced nephrotoxicity [9]. A number of reports have shown that some endogenous or exogenous antioxidants can resist oxidative stress and relieve the nephrotoxicity of GEN [10,11]. Apoptosis, necroptosis, and inflammation [9,12] all play a pivotal role in its toxicological process. However, ferroptosis, a modulated form of cell death characterized by increased lipid peroxidation due to the lack of glutathione peroxidase 4 (GPX4) activity, has not been reported in GEN-induced nephrotoxicitiy.

In recent years, epigallocatechin gallate (EG), the active ingredient in green tea, has been focused on for its excellent antioxidant, anti-inflammatory, antitumor, and antibacterial effects. Studies have demonstrated that EG can alleviate diseases such as acute radiation esophagitis [13], ionizing radiation-induced intestinal epithelial cell death [14], pneumonia caused by coronavirus disease 2019 (COVID-19) [15], and diabetes [16]. In the studies of kidney disease, EG has also been shown to protect against the renal damage caused by cisplatin [17], contrast agents [18], and cyclosporine [19], as well as diabetic nephropathy [20]. In addition, Ahmed et al. [21] also confirmed the protective effect of EG on GEN nephrotoxicity in rats, but the specific mechanism is still unclear. Therefore, the current study further investigates the protective effect of EG on GEN nephrotoxicity through in vitro and in vivo experiments and explores its protective effect on oxidative stress and programmed cell death induced by GEN.

## 2. Results

### 2.1. EG Decreased GEN-Induced Nephrotoxicity in Sprague–Dawley (SD) Rats

**Figure 1:**
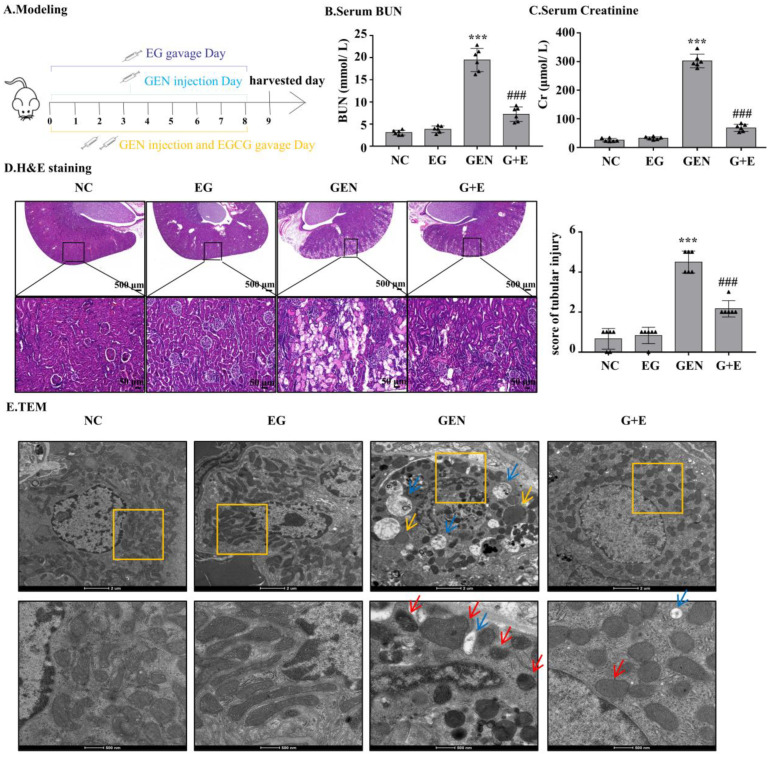
Epigallocatechin gallate (EG) reduced gentamicin (GEN)-induced nephrotoxicity in Sprague–Dawley (SD) rats. (**A**) The therapy design in different groups. SD rats were given either 100 mg/kg GEN by intraperitoneal injection or 100 mg/kg EG by oral administration daily. (**B**) Serum BUN. (**C**) Serum creatinine. (**D**) Representative HE staining images of the rat kidney sections were observed under a bright field microscope. Scale bars = 500 μm and 50 μm. In vivo data was expressed as mean ± SEM of six rats. (**E**) The ultrastructural changes of kidney were observed by transmission electron microscopy. The red arrow indicated mitochondrial cristae disappearance and outer membrane rupture. The yellow arrows indicated the increased peroxisome and the blue arrows indicated the increased autophagic vacuoles. Scale bars = 2 μm and 500 nm. *** *p* < 0.001 vs. the normal group; ### *p* < 0.001 vs. GEN group.

GEN injection for eight continuous days caused increased levels of serum BUN and serum creatinine, which were significantly attenuated by EG treatment (Figure 1B,C). Intragastric administration of EG alone did not affect the levels of serum BUN and serum creatinine. HE-stained sections showed that compared with the normal group, the renal tubule dilated and epithelial cells exfoliated significantly in the GEN group. The degree of renal tubular damage was significantly reduced after EG treatment (Figure 1D). The ultrastructure observed by transmission electron microscopy showed that the nuclear membrane and nucleolus of tubular cells were broken, the number of peroxisome and autophagic vacuoles increased, the number of mitochondria decreased and the morphology of mitochondria changed. At high magnification, it was observed that the mitochondrial bilayer structure was destroyed and the mitochondrial crista disappeared in the GEN group, whereas EG treatment alleviated this abnormality (Figure 1E).

### 2.2. EG Attenuated GEN-Induced Cell Apoptosis and Ferroptosis In Vivo

**Figure 2:**
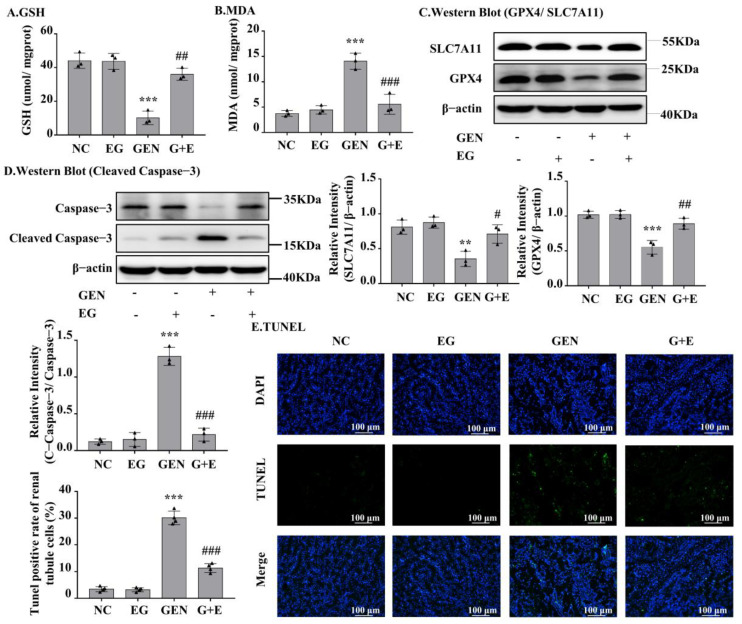
EG significantly decreased GEN-induced renal cell apoptosis and ferroptosis in SD rats. (**A**) Glutathione (GSH) level in kidney tissue samples. (**B**) Malondialdehyde (MDA) level in kidney tissue samples. (**C**) The Western blot analysed the levels of SLC7A11 and glutathione peroxidase 4 (GPX4) in SD rat kidney. (**D**) The Western blot analysed the level of cleaved-caspase-3 protein in SD rat kidney. (**E**) TUNEL staining of the rat kidney. Scale bars = 100 μm. In vivo data was expressed as mean ± SEM of three or four rats. ** *p* < 0.01, *** *p* < 0.001 vs. control group; # *p* < 0.05, ## *p* < 0.01, ### *p* < 0.001 vs. GEN group.

GEN induced an increase in malondialdehyde (MDA) and a decrease in glutathione (GSH) in kidney tissue, and EG effectively prevented these changes (Figure 2A,B). As shown in Figure 2C, GEN obviously decreased the protein levels of GPX4 and SLC7A11, which were rescued by EG. The Western blot result showed that the cleaved-caspase-3 in renal samples of GEN-treated rats was significantly decreased by the treatment of EG (Figure 2D). TUNEL staining of the kidney tissue sections showed that the cell death induced by GEN was attenuated by the treatment of EG (Figure 2E).

### 2.3. EG Decreased the Toxicity and Peroxidation of Lipid of GEN in Renal Cells

**Figure 3:**
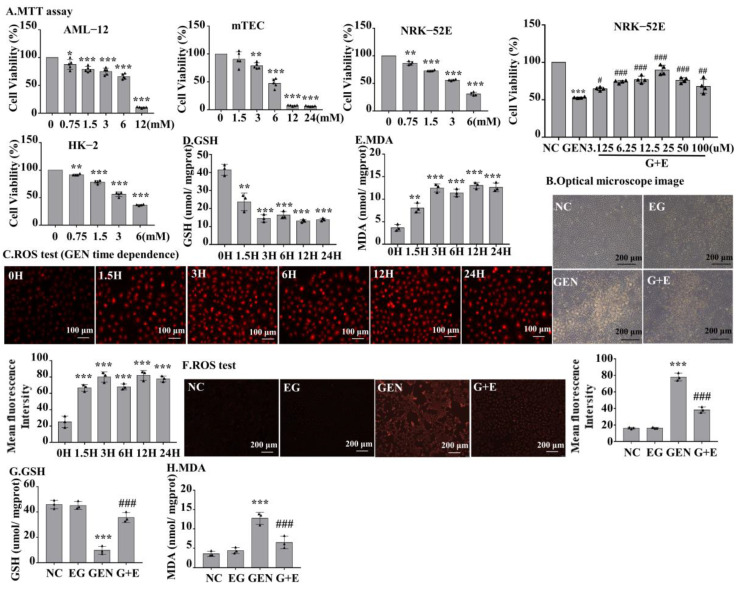
The effect of GEN on cell viability of different cells and the level of ROS, GSH, and MDA in normal rat kidney cell line 52E (NRK-52E) cells. (**A**) An MTT assay was used to determine the effects of GEN at different concentrations on the viability of alpha mouse liver 12, mouse renal tubular epithelial cells, NRK-52E, and human proximal tubular epithelial cell line cells. The optimal protective concentration of EG was detected by the MTT experiment. (**B**) Observed by light microscope, the damage of cells was serious in GEN (3 mM) treatment, but reduced in the EG (25 μM) and GEN (3 mM) combined treatment groups. Scale bars = 200 μm. (**C**) Dihydroethidium (DHE) staining was used to detect the ROS level in NRK-52E cells. Scale bars = 100 μm. (**D**) GSH level in NRK-52E cells. (**E**) MDA level in NRK-52E cells. (**F**) DHE staining results showed that EG (25 μM) could reduce the increase of ROS in NRK-52E cells induced by GEN (3 mM). (**G**) GSH. (**H**) MDA. In vitro data was expressed as mean ± SEM of three independent experiments. * *p* < 0.05, ** *p* < 0.01, *** *p* < 0.001 vs. control group; # *p* < 0.05, ## *p* < 0.01, ### *p* < 0.001 vs. GEN group.

MTT was used to detect the toxic effects of GEN to different types of cells at the indicated concentrations. The result demonstrated that GEN decreased cell viability concentration-dependently. When cells were stimulated with 3 mM GEN, the viability of normal rat kidney cell line 52E (NRK-52E) and human proximal tubular epithelial cell line (HK-2) cells decreased by approximately 50%. However, the viability of alpha mouse liver 12 (AML-12) and mouse renal tubular epithelial cells (mTEC) decreased by only 25% (Figure 3A), suggesting the toxic sensitivity of GEN to rat and human renal tubular cells. To investigate the role of EG on GEN-induced cytotoxicity, NRK-52E and HK-2 cells were stimulated with 3 mM GEN and different concentrations of EG, and it was found that 25 μM EG had the best protective effect (Figure 3A and Figure S1A). The observation of cell morphology under light microscope revealed that EG treatment improved the damage caused by GEN (Figure 3B). NRK-52E cells were treated with 3 mM GEN for 1.5 H, 3 H, 6 H, 12 H, and 24 H, respectively. Dihydroethidium (DHE) staining results showed that the intracellular ROS levels in NRK-52E cells increased rapidly at the initial stage of GEN stimulation and remained high and stable from 1.5 H to 24 H (Figure 3C). Moreover, the trends of changes of GSH and MDA in NRK-52E cells were similar (Figure 3D,E). When treated with 25 μM EG, it effectively reduced the intracellular ROS level (Figure 3F) and prevented the changes of GSH and MDA (Figure 3G,H) in GEN-treated cells.

### 2.4. EG Reduced the Apoptosis and Ferroptosis of NRK-52E Cells Induced by GEN

**Figure 4:**
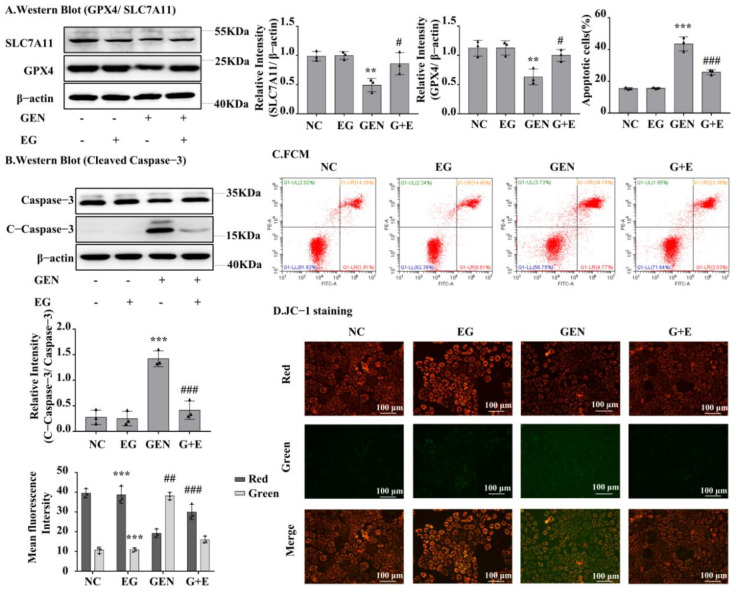
EG reduced GEN-induced apoptosis and ferroptosis of NRK-52E cells. (**A**) The Western blot analysed the levels of the GPX4 and SLC7A11 in NRK-52E cells. (**B**) The Western blot analysed the level of the cleaved-caspase-3 in NRK-52E cells. (**C**) Apoptosis of NRK-52E cells was analysed by flow cytometry. (**D**) JC-1 staining showed the changes of mitochondrial membrane potential in NRK-52E cells. Enhanced red fluorescence indicated formation of JC-1 aggregates, suggesting a relatively intact mitochondrial membrane. Enhanced green fluorescence indicated the production of JC-1 monomer, suggesting the destruction of mitochondrial membrane. Scale bars = 100 μm. In vitro data was expressed as mean ± SEM of three independent experiments. ** *p* < 0.01, *** *p* < 0.001 vs. control group; # *p* < 0.05, ## *p* < 0.01, ### *p* < 0.001 vs. GEN group.

Western blot analysis showed that GEN could downregulate GPX4 and SLC7A11 expression in NRK-52E cells, whereas EG could restore GPX4 and SLC7A11 expression (Figure 4A). GEN obviously increased the protein levels of cleaved-caspase-3, which were rescued by EG (Figure 4B). Consistently, the flow cytometry analysis showed that GEN increased the number of apoptotic cells, whereas it decreased after EG treatment (Figure 4C). In addition, JC-1 results showed an increase in green fluorescence and a decrease in red fluorescence when treated with GEN, indicating a decrease in mitochondrial membrane potential and an increase in the fraction of early mitochondrial apoptotic cells. However, the combined use of EG reversed this phenomenon (Figure 4D).

### 2.5. EG-Activated Nuclear Factor Erythroid 2-Related Factor 2 (Nrf2) Signaling

**Figure 5:**
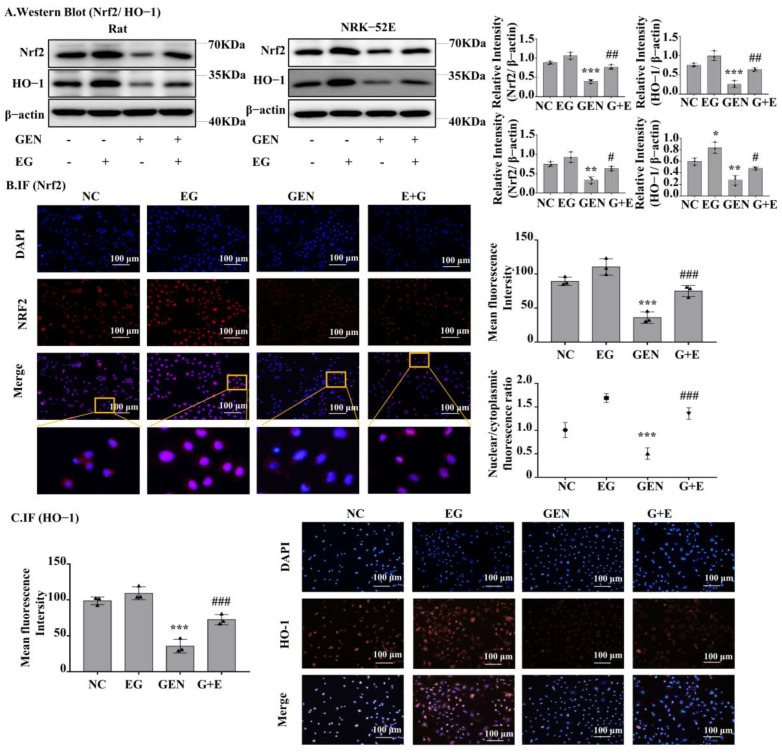
EG reversed GEN-induced oxidative stress in NRK-52E. (**A**) Western blot analysis of nuclear factor erythroid 2-related factor 2 (Nrf2) and heme oxygenase-1 (HO-1) in the renal cortex of SD rats and NRK-52E cells. EG (25 μM) treatment increased the levels of Nrf2 and HO-1. (**B**) Immunofluorescence analysis of Nrf2 expression in NRK-52E cells. Scale bars = 100 μm. (**C**) Immunofluorescence analysis of HO-1 expression in NRK-52E cells. Scale bars = 100 μm. In vitro data was expressed as mean ± SEM of three independent experiments. * *p*< 0.05, ** *p* < 0.01, *** *p* < 0.001 vs. control group; # *p* < 0.05, ## *p* < 0.01, ### *p* < 0.001 vs. GEN group.

Western blot analysis showed that Nrf2 and heme oxygenase-1 (HO-1) protein levels in the GEN-treated group were significantly lower than the control group and EG can increase their expression to a normal level (Figure 5A). The results of RT-qPCR also showed the mRNA levels of Nrf2 and HO-1 in GEN-treated NRK-52E cells and rat kidney tissue were significantly downregulated by GEN and they could be partially restored by the treatment of EG (Figure S1B). In addition, the single use of the EG increased the protein levels of Nrf2 and HO-1 compared to the control group. Immunofluorescence staining of Nrf2 and HO-1 in NRK-52E cells showed a similar tendency (Figure 5B,C). Meanwhile, EG treatment enhanced the translocation of Nrf2 to the nucleus (Figure 5B).

### 2.6. Knockdown of Nrf2 Abrogated the Protective Effects of EG

**Figure 6:**
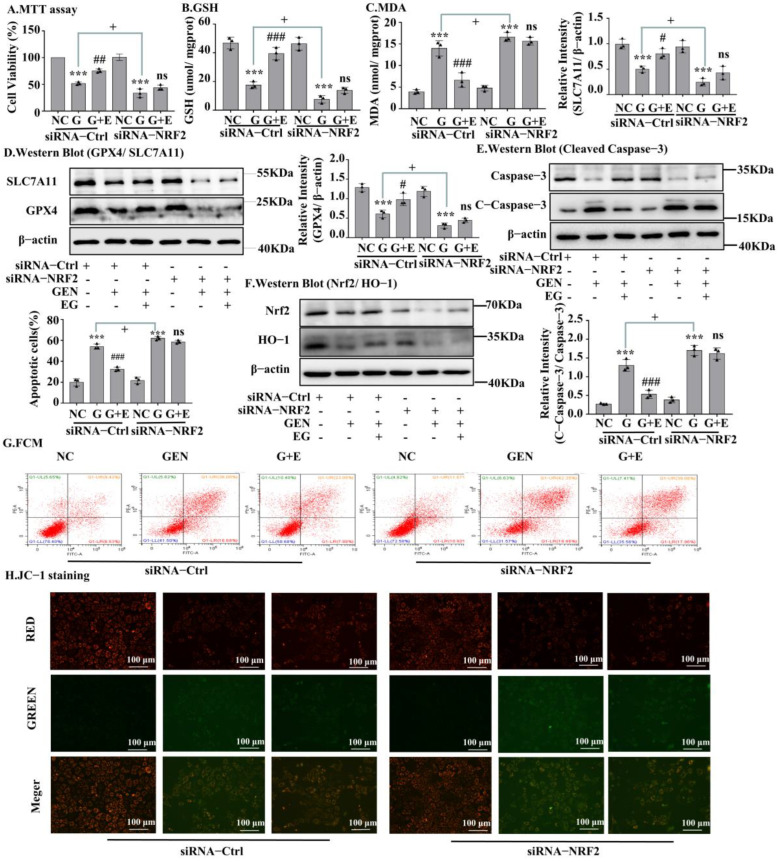
The silent Nrf2 prevented EG from improving GEN-induced apoptosis and ferroptosis. (**A**) NRK-52E cells were treated with siRNA-Nrf2 or negative control siRNA (siRNA-Ctrl) for 24 H. Cell viability was determined by using MTT assay, by using NC group as control. (**B**) GHS level in NRK-52E cells. (**C**) MDA level in NRK-52E cells. (**D**) Western blot analysis of GPX4 and SLC7A11 in NRK-52E cells. (**E**) Western blot analysis of Nrf2 and HO-1 in NRK-52E cells. (**F**) Western blot analysis of cleaved-caspase-3 in NRK-52E cells. (**G**) Apoptosis of NRK-52E cells was analysed by flow cytometry. (**H**) The JC-1 staining of NRK-52E cells showed that after NRK-52E cells silenced Nrf2, EG (25 μM) combined with GEN (3 mM) did not improve the red fluorescence reduction and green fluorescence enhancement caused by GEN compared with GEN (3 mM) alone. Scale bars = 100 μm. In vitro data was expressed as mean ± SEM of three independent experiments. *** *p* < 0.001 vs. control group; # *p* < 0.05, ## *p* < 0.01, ### *p* < 0.001 vs. GEN group; + *p* < 0.05 siRNA-Ctrl GEN group vs. siRNA-Nrf2 GEN group.

A Western blot assay confirmed the silencing of Nrf2 by siRNA transfection (Figure 6F and Figure S1C). MTT results in NRK-52E cells showed that the protective effect of EG on cell viability (Figure 6A) and on the decrease of GSH and the increase of MDA induced by GEN was weakened (Figure 6B,C), after Nrf2 knockdown. When knocking down Nrf2, there were no statistical differences in the levels of GPX4 and SLC7A11 protein between the EG + GEN group and the GEN group (Figure 6D). Additionally, the effect of EG on reducing the increase of cleaved-caspase-3 level caused by GEN also disappeared. These results indicated that the knockdown of Nrf2 made EG weaken its protection against GEN (Figure 6E). This was also confirmed by the results of cell flow cytometry (Figure 6G) and JC-1 stain (Figure 6H and Figure S1D).

### 2.7. EG Interacted with Rat Kelch-like ECH-Associated Protein 1 (KEAP1) Protein

**Figure 7:**
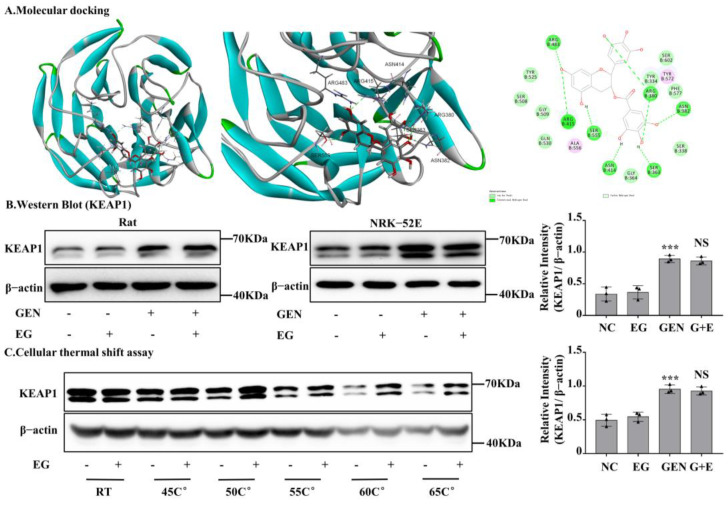
EG stabilized kelch-like ECH-associated protein 1 (KEAP1) in NRK-52E cells and molecular docking analysis. (**A**) Molecular docking model between EG and human KEAP1 protein. EG formed 10 hydrogen bonds with the side chains of R483, R415, S555, N414, S363, R380, and N382. (**B**) The protein level of KEAP1 was analysed by Western blot. (**C**) NRK-52E cells treated with EG (25 μM) for 2 H were collected, put through the freeze–thaw process three times, and heated at the corresponding temperature for 10 min. The Western blot analysed the supernatant protein. In vitro data was expressed as mean ± SEM of three independent experiments. *** *p* < 0.001 vs. control group; NS vs. GEN group.

After silencing Nrf2 in NRK-52E cells, the protective effect of EG against EGN-induced nephrotoxicity was weakened. In numerous reported studies, KEAP1, a conjunctive protein, binds to the antioxidant enzyme transcription factor Nrf2. Moreover, KEAP1 is easily modified by electrophilic substances [22]. In order to further explore whether EG affects Nrf2 protein activity by binding to KEAP1 protein, we detected the binding of EG to KEAP1 protein by means of molecular docking. It predicted that EG could form nine hydrogen bonds with the amino acid residues of R483, R415, S555, N414, S363, R380, and N382 of human KEAP1 protein (Figure 7A). BLAST results showed that rat KEAP1 protein sequence was highly homologous to human KEAP1 and the above key amino acid residues also existed in rat KEAP1 protein (Figure S2A,B). Therefore, it suggested that EG might be a regulator of the KEAP1 protein in rats. In order to further explore the effect of EG on the KEAP1, we detected the protein level of KEAP1 in different treatment groups. Moreover, the results showed that GEN could significantly increase the expression of KEAP1, but there was no change after the combined use of EG (Figure 7B). The results of cell thermal stability showed that EG could stabilize the protein level of rat KEAP1 in NRK-52E cell lysates under the temperature of 25 °C, 45 °C, 50 °C, 55 °C, 60 °C, and 65 °C (Figure 7C).

### 2.8. EG Showed No Significant Effect on the Antimicrobial Activity of GEN against E. coli

**Figure 8:**
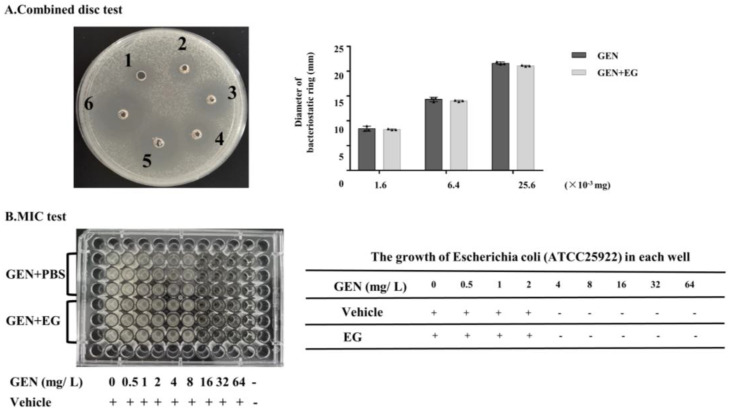
The effect of EG on the antimicrobial activity of GEN. (**A**) Combined disc test was used to detect the effect of EG (25 μM) on the inhibitory ring of GEN against *Escherichia coli* (*E. coli*). (**B**) A minimal inhibitory concentration (MIC) test was used to analyse the impact of EG (25 μM) on the antibacterial activity of GEN to *E. coli* in the liquid culture medium. A “+” indicates bacterial growth and a “−” indicates no bacterial growth. In vitro data was presented as the mean ± SEM of three independent experiments.

EG was used concomitantly with GEN in the culture medium containing *E. coli* to verify whether it affects the antibacterial activity of GEN. The combination disc test indicated that there was slight difference in the diameters of the inhibitory zone between the group treated with GEN and GEN + EG (Figure 8A). Moreover, the measurement of the MIC showed that the MIC of GEN to *E. coli* is 4 mg/L and EG had no impact on this MIC (Figure 8B).

## 3. Discussion

The most common adverse drug reaction to aminoglycoside drugs, especially GEN, is nephrotoxicity [23]. The GEN-induced generation of oxygen free radicals (superoxide, hydroxyl radicals, hydrogen peroxide, etc.) and GEN-induced reduction of cellular antioxidant capacity are considered as the main causes of nephrotoxicity [24]. In the present study, we found that 3 mM GEN could induce the increase of intracellular ROS level as early as 1.5 H since the treatment. DHE staining also showed that the intracellular ROS level reached a high level at 1.5 H after GEN treatment, but there was no significant change in intracellular ROS level at 3 H, 6 H, 12 H, and 24 H after GEN treatment. The significant increase of ROS levels at the early stage of GEN treatment indicates that oxidative stress is a key node in the toxicological process. In the present study, we detected the levels of protein markers of apoptosis and ferroptosis, which were aggravated. EG has been reported to be an effective scavenger of oxygen free radicals and to protect against oxidative damage from many insults [25,26]. Therefore, the protective effect of EG on GEN nephrotoxicity is investigated in both in vivo and in vitro models, in the present study. 

Recently, Hebatalla et al. studied the nephrotoxicity of GEN in rats and found that the use of candesartan and EG, respectively, could improve the renal damage, and the combined use enhanced the protective effect [21]. In the study, it was found that EG can reduce levels of P38MAPK and P65 in kidney injury. In addition, EG was found with a protective effect against GEN-induced ototoxicity. Zong et al. [27] proved in a zebrafish model treated with GEN that EG could reduce the level of oxidative stress and the apoptosis of cochlear hair cells. Jiang et al. [28] proved that EG can reduce GEN-induced oxidative stress in cochlear hair cells from newborn mouse cochlea, and the inhibition of STAT1 signaling pathway could be the mechanism of EG [29]. However, it is not clear whether EG plays an antioxidant role in combating GEN-induced renal injury and whether the oxidative stress induced by GEN can trigger apoptosis and ferroptosis.

Xie et al. [14] once found that EG could play an important protective role in radiation-induced intestinal injury through Nrf2 and its downstream signals. The protective effect of EG would be eliminated by pretreatment with ML385, an inhibitor of Nrf2 [14,30]. In our study, the protein levels of renal Nrf2 and HO-1 significantly increased when EG was used alone, but decreased by the single GEN treatment. Immunofluorescence results also suggested that EG could promote Nrf2 translated into the nucleus. In order to determine whether the protective effect of EG depends on Nrf2, we knocked down Nrf2 by the transfection of siRNA targeted to Nrf2 (the protein levels were reduced by about 3/4). After the knockdown, the toxicity of GEN was aggravated, compared with the negative control. Nrf2 knockdown weakened the antioxidant ability of EG, as well as the regulatory ability of GSH, MDA, and HO-1. More importantly, when Nrf2 was knocked down, EG had a weakened protective effect against GEN-induced cell apoptosis and ferroptosis. Based on these data, the Nrf2 signaling pathway is important for the protective effect of EG against GEN-induced nephrotoxicity.

Moreover, in this study the result of molecular docking indicated that EG had a binding effect on human KEAP1, and the sequence of rat KEAP1 protein is homologous to humans, indicating that the corresponding binding site of EG may also exist in rat protein. Shanmugam et al. [31] also predicted that EG can bind to human KEAP1 protein to activate Nrf2 to eliminate fluorine-induced pulmonary oxidative stress. When rat renal tubular cells were incubated with EG, KEAP1 became more stable at high temperatures as shown by the thermal shift assay test in our study. The predicted binding site of EG is in the range from TYR334 to PHE575 of KEAP1, which fall in the domain of kelch (from amino acid site of 309 to 624). Nrf2 can bind to KEAP1 via the kelch domain [22]. Therefore, EG may be embedded in this region and lead to the dissociation of KEAP1 and Nrf2, followed by the translocation of Nrf2 into the nucleus [32]. There are other mechanisms of action between EG and KEAP1,for example EG and KEAP1 proteins form the EG-KEAP1 adduct, which blocks the degradation of Nrf2 [33]. Under normal conditions, EG did not affect the protein expression of KEAP1, which was also found in the study of Shanmugam et al. [31]. As a key transcription factor for the antioxidant system, Nrf2 modulates the expression of antioxidant factors such as HO-1 and GPX4 through antioxidant response elements (ARE) [34]. Moreover, in the present study we found that GEN significantly increased the expression of KEAP1 that might lead to the cytoplasmic localization and the degradation of Nrf2. The potential regulatory mechanism of GEN to KEAP1 is currently unknown and needs to be further studied.

However, this study also has some limitations. First, NRF2 was not knocked down in vivo; therefore, the protective effect of EG against GEN-induced nephrotoxicity in vivo needs further validation. Secondly, although the apoptosis and ferroptosis of renal cells is the main pathological manifestation of GEN-induced nephrotoxicity, other forms of cell death, such as programmed necrosis and pyroptosis, have not been studied in our models [35]. Future research will continue to revolve around these problems.

## 4. Materials and Methods

### 4.1. Reagents and Materials

Fetal bovine serum (FBS), DMEM, and other cell culture reagents were purchased from Invitrogen (Carlsbad, CA, USA). GEN was obtained from Shandong Cisen Pharmaceutical (Anhui, China). EG was purchased from meilunbio (Liaoning, China). Antibody-targeting Nrf2 (bs-1074R; WB: 1:500; IHC: 1:200) was obtained from Bioss (Beijing, China). Antibody-targeting SLC7A11 (26864-1-AP; WB: 1:500) and HO-1/HMOX1 (10701-1-AP; WB: 1:1000; IHC: 1:200) were obtained from Proteintech (Wuhan, China). Antibody-targeting GPX4 (JP47937; WB: 1:500) was provided by ELISA LAB (Wuhan, China). The anti-β-actin antibody (AC004; WB: 1:1000) was purchased from ABclonal (Wuhan, China). The anti-caspase3/ cleaved-caspase-3 antibody (WL02117,1:500) was obtained from Wanlei (Shenyang, China). Serum BUN and serum creatinine assay kits were obtained from Nanjing Jiancheng Bioengineering Institute (Nanjing, China).

### 4.2. Animals and Drug Administration

Male SD rats (150~200 g) were purchased from the Experimental Animal Center of Anhui Medical University. The rats were kept under standard conditions of 22 °C and free of disease, and had free access to standard food and water. All animal experiments were conducted in accordance with the regulations on the control of laboratory animals promulgated by the State Science and Technology Commission. All animal experiments were approved by the Animal Experiment Ethics Committee of Anhui Medical University (LLSC20180319).

Before the experiments, 24 rats were randomly divided into the following four groups: control group rats were given the same amount of normal saline intraperitoneally or distilled water by gavage; EG group rats were given 100 mg/kg EG solely by gavage; GEN group rats were intraperitoneally injected with 100 mg/kg GEN; GEN + EG group rats were intraperitoneally injected with 100 mg/kg GEN followed by the 100 mg/kg EG by the oral administration. After the injection or oral administration of these drugs for eight continuous days (Figure 1A), the animals were anesthetized. The serum was separated from collected blood samples, and stored at −80 °C to detect serum BUN and serum creatinine. The kidney tissue was incised and kept at −80 °C for the following tests.

### 4.3. Hematoxylin and Eosin Staining and TUNEL Assay

The middle part of the rat kidney was fixed in the paraformaldehyde solution. The paraffin blocks embedded with the middle part of the kidney were cut into 5 μm thin slices and dried in the oven at 65 °C for 1 H. Subsequently, they were dewaxed with xylene, dewaxed with continuously diluted ethanol, stained with hematoxylin, washed with tap water, differentiated with differentiation solution, and stained with anti-blue solution for 1–2 min. Tissue sections were stained with 1% eosin, dehydrated with gradient alcohol and sealed with a tablet. The renal injury score indicated the extent of tubule damage as follows: 0 = normal, 1 = 1–10%, 2 = 10–25%, 3 = 26–50%, 4 = 51–75%, 5 = 76–95%, and 6 = more than 96%. The TUNEL assay kit was obtained from Beyotime Institute of Biotechnology (Jiangsu, China) and the staining process was according to the manufacturer’s instructions. The sides were examined with the fluorescent inverted microscope.

### 4.4. Transmission Electron Microscopy

After the rats were anesthetized, tissue blocks with the size of 1 mm^3^ were taken from the renal cortex of rats and fixed with a 4 °C buffer of 2.5% glutaraldehyde. After rinsing with 0.1 M caustic soda buffer, the caustic solution was permeated with 1% osmium tetroxide for 1 H at room temperature. The tissue samples were dehydrated with gradient concentration ethanol, briefly dehydrated with 100% propylene oxide, then pre-percolated overnight with a 1:1 mixture of propylene oxide and propylene oxide on a gentle rotator. After embedding with EPON 821 and resin, the tissue samples were cut into sections with a thickness of 80 mm. After staining with lead citrate, the sections were dried and observed by transmission electron microscope (TEM, Thermo Scientific Ta1os L120C G2, Waltham, MA, USA).

### 4.5. Cell Culture

NRK-52E, HK-2, mTEC, and AML-12 cells were obtained from the National Collection of Authenticated Cell Cultures (Shanghai, China). NRK-52E, HK-2, mTEC, and AML-12 cells were cultured with DMEM/ F-12 (HyClone, Logan, UT, USA) supplemented with 10% fetal bovine serum at 37 °C in an incubator with 5% carbon dioxide humidity.

### 4.6. Cell Viability Assay

The suspension of cells was cultured in 96-well cell plates and then subjected to the following treatment. First, the cells were incubated with different concentrations of GEN (0 mM, 0.75 mM, 1.5 mM, 3 mM, 6 mM). Secondly, the cells were treated with different concentrations of EG or GEN. Thirdly, the cells were transfected with Nrf2-siRNA followed by the treatment of EG or GEN. After the treatment of drugs for 24 H, the absorbance was determined by SpectraMax multimode microplate (Molecular Devices, SpectraMax iD3, USA) at 490 nm OD after being incubated with 100 μL MTT for 4 H and then treated with DMSO at 37 °C for 30 min.

### 4.7. DHE Staining and Detection of GSH and MDA Levels

DHE obtained from Beyotime Institute of Biotechnology (Jiangsu, China) was used to detect ROS levels in NRK-52E cells. After GEN treatment for 1.5 H, 3 H, 6 H, 12 H, 24 H or concurrent use of EG, the NRK-52E cells were incubated with a solution containing 5 μM DHE for 30 min at 37 °C. After washing with PBS, the plate was visualized on the inverted fluorescent microscope.

The kit obtained from Nanjing Jiancheng Bioengineering Institute (Nanjing, China) was used to detect the GSH and MDA levels in kidney tissue samples and cells. The individual contents of the GSH and MDA for the different treatments were determined from the OD values at 405 nm and 523 nm, following the manufacturer’s instructions. The total protein content was detected by using the BCA protein concentration assay kit purchased from Beyotime Biotechnology (Shanghai, China).

### 4.8. JC-1

The suspension of NRK-52E cells were evenly cultured in 12-well cell plates. After the incubation of drugs, the supernatant was removed, and 10 μL JC-1 (200 μM) was added to each well and incubated at 37 °C for 20 min. After washing three times with PBS, the plate was visualized on the inverted fluorescent microscope.

### 4.9. Assessment of Apoptosis by Flow Cytometry

The suspension of NRK-52E cells were cultured in 6-well cell plates. After different treatment, the cells were collected and then dyed with annexin V-fluorescein isothiocyanate (FITC) and propidium iodide (PI) following the manufacturer’s instructions and the PI and FITC positive cells were considered apoptotic cells.

### 4.10. Immunofluorescence Staining

NRK-52E cells were cultured in 12-well cell plates with the cover slides at the bottom and treated with different drugs. The slides were then taken out and fixed with acetone. After washing with PBS, the slides were blocked with 10% goat serum solution to avoid nonspecific staining. The cell slides were incubated with a polyclonal primary antibody against Nrf2 (1:200) overnight at 4 °C. Then, the cell slides were incubated with secondary antibody for 1 H at room temperature and the nucleus was dyed with 4, 6-diamidino-2-phenylindole (DAPI; Beyotime Biotechnology, Shanghai, China). Finally, the cell slides were visualized on the fluorescent inverted microscope and the quantitative analysis of IF images were conducted by using Image J software.

### 4.11. Transfection of Small Interfering RNA (siRNA)

NRK-52E cells were grown in 6-well plates to approximately 60% confluence. The cells were transfected with Lipofectamine 2000 following the manufacturer’s instructions. The rat-specific siRNA for Nrf2 was manufactured by GenePharma biology (Shanghai, China) and the sequences are as follows: 5′-GGGUAAGUCGAGAAGUGUUTT-3′ and 5′-AACACUUCUCGACUUACCTT-3′ [36]. GenePharma Biology (Shanghai, China) designed and manufactured the rat negative control siRNA (NC siRNA) with the following sequence: 5′-UUCUCCGAACGUGUCACGUTT-3′ and 5′-ACGUGACACGUUCGGAGAATT-3′.

### 4.12. Western Blot

The protein samples were obtained from the renal cortex or the cells by using RIPA buffer (Beyotime, Jiangsu, China) containing PMSF. Then, the protein samples were transferred to polyvinylidene difluoride (PVDF) membranes after separated by 10% or 12% sodium dodecyl sulfate polyacrylamide gel electrophoresis (SDS-PAGE), incubating with 5% milk in TBS for 1–2 H at room temperature to block nonspecific binding. The protein bands were incubated with primary antibodies overnight at 4 °C, subsequently incubated with the secondary antibodies for 1–2 H at 37 °C, and visualized with an ECL chemiluminescent kit (ECL-plus; Thermo Fisher Scientific, Pittsburgh, PA, USA) after washing with TBST (0.1% Tween 20).

### 4.13. Molecular Docking

Molecular docking was used to investigate the possible binding effect between EG and KEAP1/ Nrf2 and performed by using the Discovery Studio 2017 program. The structure of KEAP1 protein was obtained from the Protein Data Bank (PDB, ID: 7K2H). The drug structure of EG was obtained from Meilunbio (Liaoning, China) product information. Prior to docking, all water molecules and ligand structures were removed from the initial structure of the KEAP1 protein [37]. The docking program was performed by CDOCKER module. Unless otherwise specified, the interconnection parameters were set to default values.

### 4.14. Thermal Shift Assay

NRK-52E cells were seeded on 6-well plates. One group was incubated with EG for 2 H, and the other group was incubated with blank medium as a control. Then, the two groups of cells were collected and rinsed with PBS containing protease inhibitor. Subsequently, the cell suspension was repeatedly frozen with liquid nitrogen three times. After mixing well, each group was divided into six equal parts. One for each group was kept at room temperature and the rest were added to 0.2 mL PCR tubes. After gradient heating (45 °C, 50 °C, 55 °C, 60 °C, and 65 °C), the samples were cooled at room temperature for 3 min and centrifuged for 15 min at 20,000 rcf. Finally, the supernatant was taken for protein denaturation and analysed by Western blot [38].

### 4.15. Combined Disc Test and MIC Determination

Escherichia coli (*E. coli*) ATCC25922 was coated on the LB broth agar medium at a concentration of 1 × 10^6^ CFU/ mL evenly. After six holes are drilled with an AGAR gel puncher, we added the following drug to each hole: GEN (25.6 × 10^−3^ mg); GEN (25.6 × 10^−3^ mg) + EG; GEN (6.4 × 10^−3^ mg); GEN (6.4 × 10^−3^ mg) + EG; GEN (1.6 × 10^−3^ mg); and GEN (1.6 × 10^−3^ mg) + EG. After incubating for 24 H at 37 °C, the diameter of inhibitory ring was measured. The *E. coli* cultured in Luria–Bertani (LB) medium was seeded onto 96-well plates. The 96-well plates containing the *E. coli* were divided into two groups. The GEN group was added with different concentrations of GEN (0, 0.5, 1, 2, 4, 8, 16, 32, and 64 mM) and the GEN + EG group added the same concentrations of GEN and EG (25 μM). Finally, the turbidity of the two groups was compared after incubation for 24 H at 37 °C [39].

### 4.16. Statistical Analysis

Data were expressed as the mean ± SEM. Statistical significance was analysed by using Tukey post hoc after tests one-way analysis of variance (ANOVA) by using GraphPad Prism 5 software (GraphPad, La Jolla, CA, USA) and the differences were considered significant where *p* < 0.05.

## 5. Conclusions

In this study, we found that cellular ROS shortly increased after GEN treatment and remained high and stable. GEN-induced nephrotoxicity in rats is associated with increased apoptosis of renal tubule cells. Moreover, our results demonstrated for the first time that ferroptosis played an important role in GEN-induced nephrotoxicity. EG can alleviate GEN-induced nephrotoxicity both in vivo and in vitro. Moreover, we suggested that EG suppressed GEN-induced apoptosis and ferroptosis through the Nrf2/ HO-1 pathway.

## Data Availability

The data that support the findings of this study are available from the corresponding author upon reasonable request.

## Supplementary Materials

The following are available online at https://www.mdpi.com/article/10.3390/molecules27238564/s1, Figure S1: EG could ameliorate the decreased HK-2 cell activity induced by GEN. Figure S2: Homology analysis of human KEAP1 protein and SD rat KEAP1 protein.

## Sample Availability

Samples of the compounds are not available from the authors.

## Abbreviations

GEN: gentamicin; EG, epigallocatechin gallate; SD rat, sprague dawley rat; BUN, blood urea nitrogen; TUNEL, TdT-mediated dUTP Nick-End Labeling; GSH, glutathione; MDA, malondialdehyde; GPX4, glutathione peroxidase 4; SLC7A11, recombinant solute carrier family 7; Nrf2, nuclear factor erythroid 2-related factor; HO-1, heme oxygenase-1; siRNA, small interfering RNA; KEAP1, kelch-like ECH-associated protein 1; FBS, fetal bovine serum; NRK-52E, normal rat kidney cell line 52E; HK-2, human proximal tubular epithelial cell line cells; mTEC, mouse renal tubular epithelial cells; AML-12, alpha mouse liver 12; ROS, reactive oxygen species; RT-qPCR, quantitative real time PCR.

## References

[1] Morales-Alvarez M.C. (2020). Nephrotoxicity of Antimicrobials and Antibiotics. Adv. Chronic Kidney Dis..

[2] Laorodphun P., Cherngwelling R., Panya A., Arjinajarn P. (2022). Curcumin protects rats against gentamicin-induced nephrotoxicity by amelioration of oxidative stress, endoplasmic reticulum stress and apoptosis. Pharm. Biol..

[3] Quiros Y., Vicente-Vicente L., Morales A.I., Lopez-Novoa J.M., Lopez-Hernandez F.J. (2011). An integrative overview on the mechanisms underlying the renal tubular cytotoxicity of gentamicin. Toxicol. Sci..

[4] Cui J., Tang L., Hong Q., Lin S., Sun X., Cai G., Bai X.Y., Chen X. (2019). N-Acetylcysteine Ameliorates Gentamicin-Induced Nephrotoxicity by Enhancing Autophagy and Reducing Oxidative Damage in Miniature Pigs. Shock.

[5] Randjelovic P., Veljkovic S., Stojiljkovic N., Sokolovic D., Ilic I. (2017). Gentamicin nephrotoxicity in animals: Current knowledge and future perspectives. EXCLI J..

[6] Sepand M.R., Ghahremani M.H., Razavi-Azarkhiavi K., Aghsami M., Rajabi J., Keshavarz-Bahaghighat H., Soodi M. (2016). Ellagic acid confers protection against gentamicin-induced oxidative damage, mitochondrial dysfunction and apoptosis-related nephrotoxicity. J. Pharm. Pharmacol..

[7] Tang Z., Song B., Zhang W., Guo L., Yuan J. (2019). Precise Monitoring of Drug-Induced Kidney Injury Using an Endoplasmic Reticulum-Targetable Ratiometric Time-Gated Luminescence Probe for Superoxide Anions. Anal. Chem..

[8] Mishima E., Sato E., Ito J., Yamada K.I., Suzuki C., Oikawa Y., Matsuhashi T., Kikuchi K., Toyohara T., Suzuki T. (2020). Drugs Repurposed as Antiferroptosis Agents Suppress Organ Damage, Including AKI, by Functioning as Lipid Peroxyl Radical Scavengers. J. Am. Soc. Nephrol..

[9] Hu Z., Zhang H., Yang S.K., Wu X., He D., Cao K., Zhang W. (2019). Emerging Role of Ferroptosis in Acute Kidney Injury. Oxidative Med. Cell. Longev..

[10] Almatroodi S.A., Almatroudi A., Khan A.A., Alhumaydhi F.A., Alsahli M.A., Rahmani A.H. (2020). Potential Therapeutic Targets of Epigallocatechin Gallate (EGCG), the Most Abundant Catechin in Green Tea, and Its Role in the Therapy of Various Types of Cancer. Molecules.

[11] Feng X., Guan W., Zhao Y., Wang C., Song M., Yao Y., Yang T., Fan H. (2019). Dexmedetomidine ameliorates lipopolysaccharide-induced acute kidney injury in rats by inhibiting inflammation and oxidative stress via the GSK-3beta/Nrf2 signaling pathway. J. Cell. Physiol..

[12] Belavgeni A., Meyer C., Stumpf J., Hugo C., Linkermann A. (2020). Ferroptosis and Necroptosis in the Kidney. Cell Chem. Biol..

[13] Zhao H., Xie P., Li X., Zhu W., Sun X., Sun X., Chen X., Xing L., Yu J. (2015). A prospective phase II trial of EGCG in treatment of acute radiation-induced esophagitis for stage III lung cancer. Radiother. Oncol..

[14] Xie L.W., Cai S., Zhao T.S., Li M., Tian Y. (2020). Green tea derivative (-)-epigallocatechin-3-gallate (EGCG) confers protection against ionizing radiation-induced intestinal epithelial cell death both in vitro and in vivo. Free Radic. Biol. Med..

[15] Chourasia M., Koppula P.R., Battu A., Ouseph M.M., Singh A.K. (2021). EGCG, a Green Tea Catechin, as a Potential Therapeutic Agent for Symptomatic and Asymptomatic SARS-CoV-2 Infection. Molecules.

[16] Mohan T., Velusamy P., Chakrapani L.N., Srinivasan A.K., Singh A., Johnson T., Periandavan K. (2017). Impact of EGCG Supplementation on the Progression of Diabetic Nephropathy in Rats: An Insight into Fibrosis and Apoptosis. J. Agric. Food Chem..

[17] Fatima S., Al-Mohaimeed N., Al-Shaikh Y., Tyagi P., Banu N., Hasan S., Arjumand S. (2016). Combined treatment of epigallocatechin gallate and Coenzyme Q10 attenuates cisplatin-induced nephrotoxicity via suppression of oxidative/nitrosative stress, inflammation and cellular damage. Food Chem. Toxicol..

[18] Palabiyik S.S., Dincer B., Cadirci E., Cinar I., Gundogdu C., Polat B., Yayla M., Halici Z. (2017). A new update for radiocontrast-induced nephropathy aggravated with glycerol in rats: The protective potential of epigallocatechin-3-gallate. Ren. Fail..

[19] Chang E.J., Mun K.C. (2004). Effect of epigallocatechin gallate on renal function in cyclosporine-induced nephrotoxicity. Transplant. Proc..

[20] Zhu Q.Q., Yang X.Y., Zhang X.J., Yu C.J., Pang Q.Q., Huang Y.W., Wang X.J., Sheng J. (2020). EGCG targeting Notch to attenuate renal fibrosis via inhibition of TGFbeta/Smad3 signaling pathway activation in streptozotocin-induced diabetic mice. Food Funct..

[21] Ahmed H.I., Mohamed E.A. (2019). Candesartan and epigallocatechin-3-gallate ameliorate gentamicin-induced renal damage in rats through p38-MAPK and NF-kappaB pathways. J. Biochem. Mol. Toxicol..

[22] Vriend J., Reiter R.J. (2015). The Keap1-Nrf2-antioxidant response element pathway: A review of its regulation by melatonin and the proteasome. Mol. Cell. Endocrinol..

[23] Pierson-Marchandise M., Gras V., Moragny J., Micallef J., Gaboriau L., Picard S., Choukroun G., Masmoudi K., Liabeuf S., French National Network of Pharmacovigilance Centres (2017). The drugs that mostly frequently induce acute kidney injury: A case—Noncase study of a pharmacovigilance database. Br. J. Clin. Pharmacol..

[24] Wang X., Asghar M. (2017). Protein disulfide isomerase regulates renal AT1 receptor function and blood pressure in rats. Am. J. Physiol. Ren. Physiol..

[25] Shi J., Zhang M., Zhang L., Deng H. (2018). Epigallocatechin-3-gallate attenuates microcystin-LR-induced apoptosis in human umbilical vein endothelial cells through activation of the NRF2/HO-1 pathway. Environ. Pollut..

[26] Zhang Y., Lin H., Liu C., Huang J., Liu Z. (2020). A review for physiological activities of EGCG and the role in improving fertility in humans/mammals. Biomed. Pharm..

[27] Zong Y., Chen F., Li S., Zhang H. (2021). (-)-Epigallocatechin-3-gallate (EGCG) prevents aminoglycosides-induced ototoxicity via anti-oxidative and anti-apoptotic pathways. Int. J. Pediatr. Otorhinolaryngol..

[28] Jiang P., Ray A., Rybak L.P., Brenner M.J. (2016). Role of STAT1 and Oxidative Stress in Gentamicin-Induced Hair Cell Death in Organ of Corti. Otol. Neurotol..

[29] Gu L.T., Yang J., Su S.Z., Liu W.W., Shi Z.G., Wang Q.R. (2015). Green Tea Polyphenols Protects Cochlear Hair Cells from Ototoxicity by Inhibiting Notch Signalling. Neurochem. Res..

[30] Zhu W., Tang H., Cao L., Zhang J., Li J., Ma D., Guo C. (2022). Epigallocatechin-3-O-gallate ameliorates oxidative stress-induced chondrocyte dysfunction and exerts chondroprotective effects via the Keap1/Nrf2/ARE signaling pathway. Chem. Biol. Drug Des..

[31] Shanmugam T., Selvaraj M., Poomalai S. (2016). Epigallocatechin gallate potentially abrogates fluoride induced lung oxidative stress, inflammation via Nrf2/Keap1 signaling pathway in rats: An in-vivo and in-silico study. Int. Immunopharmacol..

[32] Jiang Y., Yin Z., Zhao J., Sun J., Zhao D., Zeng X.A., Li H., Huang M., Wu J. (2021). Antioxidant mechanism exploration of the tripeptide Val-Asn-Pro generated from Jiuzao and its potential application in baijiu. Food Chem. Toxicol..

[33] Yang C.S., Chen T., Ho C.T. (2022). Redox and Other Biological Activities of Tea Catechins That May Affect Health: Mechanisms and Unresolved Issues. J. Agric. Food Chem..

[34] Ali F.E.M., Sayed A.M., El-Bahrawy A.H., Omar Z.M.M., Hassanein E.H.M. (2021). Targeting KEAP1/Nrf2, AKT, and PPAR-gamma signals as a potential protective mechanism of diosmin against gentamicin-induced nephrotoxicity. Life Sci..

[35] Song X., Long D. (2020). Nrf2 and Ferroptosis: A New Research Direction for Neurodegenerative Diseases. Front. Neurosci..

[36] Xu J., Lin C., Wang T., Zhang P., Liu Z., Lu C. (2018). Ergosterol Attenuates LPS-Induced Myocardial Injury by Modulating Oxidative Stress and Apoptosis in Rats. Cell. Physiol. Biochem..

[37] Sun W., Liu X., Zhang H., Song Y., Li T., Liu X., Liu Y., Guo L., Wang F., Yang T. (2017). Epigallocatechin gallate upregulates NRF2 to prevent diabetic nephropathy via disabling KEAP1. Free Radic. Biol. Med..

[38] Han S., Gao H., Chen S., Wang Q., Li X., Du L.J., Li J., Luo Y.Y., Li J.X., Zhao L.C. (2019). Procyanidin A1 Alleviates Inflammatory Response induced by LPS through NF-kappaB, MAPK, and Nrf2/HO-1 Pathways in RAW264.7 cells. Sci. Rep..

[39] Hu B.F., Gong Q., Chen S.Q., Yue L., Ma W.X., Wang F., Feng X.W., Wang J.N., Li C., Liu M.M. (2022). Protective effect of inhibiting necroptosis on gentamicin-induced nephrotoxicity. FASEB J..

