# Supplementary material for: Epigallocatechin Gallate Attenuates Gentamicin-Induced Nephrotoxicity by Suppressing Apoptosis and Ferroptosis
Source: Molecules. 2022 Dec 5;27(23):8564. doi: 10.3390/molecules27238564 (PMC9735461; doi:10.3390/molecules27238564)
Supplement: Supplementary file 1 [file molecules-27-08564-s001.zip › molecules-2050185-supplementary.pdf]

Supplementary Materials

Epigallocatechin Gallate Attenuates Gentamicin-Induced Nephrotoxicity by Suppressing Apoptosis and Ferroptosis

Lin Yue [1,†], Ya-Ru Yang [2,†], Wen-Xian Ma [1], Hong-Yan Wang [3], Qian-Wen Fan [1], Yue-Yue Wang [1], Chao Li [1], Jing Wang [1], Zi-Mu Hu [1], Xue-Fu Wang [1], Feng-He Li [1], Ming-Ming Liu [1], Juan Jin [1], Chao Shi [4,*] and Jia-Gen Wen [1,*]

1 Inflammation and Immune Mediated Diseases Laboratory of Anhui Province, Anhui Institute of Innovative Drugs, School of Pharmacy, Anhui Medical University, Hefei 230032, China
2 Department of Clinical Pharmacology, Second Hospital of Anhui Medical University, Hefei 230601, China
3 State Key Laboratory of Tea Plant Biology and Utilization, School of Tea and Food Science and Technology, Anhui Agricultural University, Hefei 230036, China
4 Department of Cardiac Surgery, The First Affiliated Hospital of Bengbu Medical College, Bengbu 233004, China
* Correspondence: wuweishichao@126.com (C.S.); jiagen168@163.com (J.-G.W.);
Tel.: Tel.: +86-0552-308635 (C.S.); +86-0551-65172131 (J.-G.W.)
† These authors contributed equally to the article.

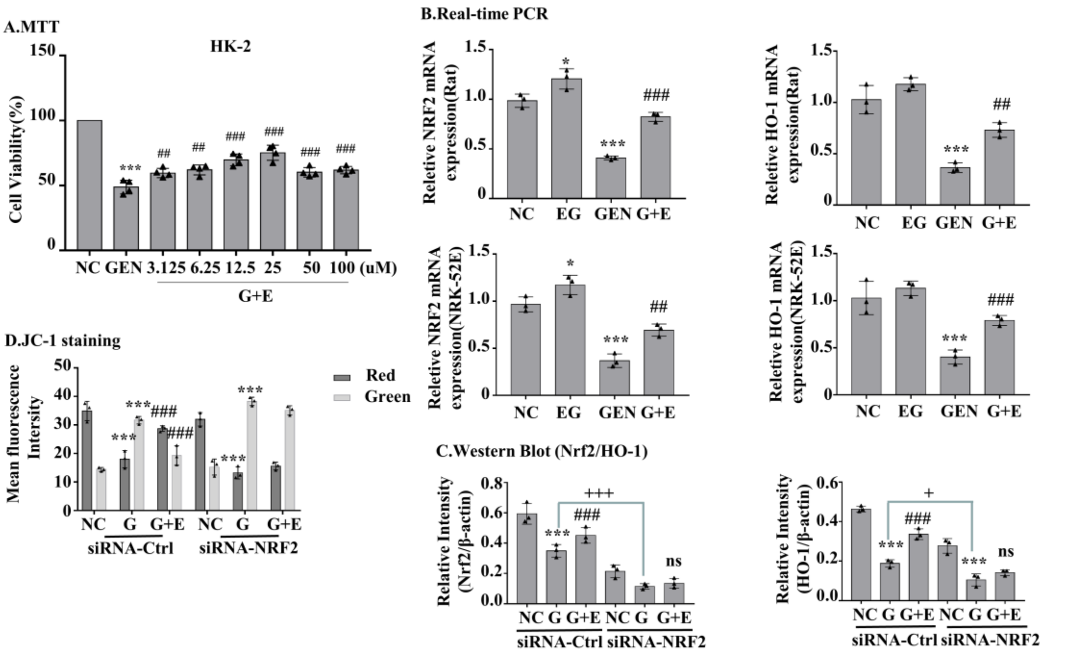

Figure S1. EG could ameliorate the decreased HK-2 cell activity induced by GEN. (A) MTT assay was used to determine the optimal protective concentration of EG on the viability of HK-2 cells. **(B)** The mRNA levels of Nrf2 and HO-1 in rat and NRK-52E cells were detected by RT-qPCR. In vivo and vitro data were expressed as mean ± SEM of three independent experiments. **(C)** Statistical analysis of western Blot (Nrf2/HO-1). **(D)** Statistical analysis of JC-1. *p<0.05, **p<0.01, ***p<0.001 vs. control group; #p<0.05, ##p<0.01, ###p<0.001 vs GEN group. +p<0.05 siRNA-Ctrl GEN group vs. siRNA-Nrf2 GEN group.

A.BLAST

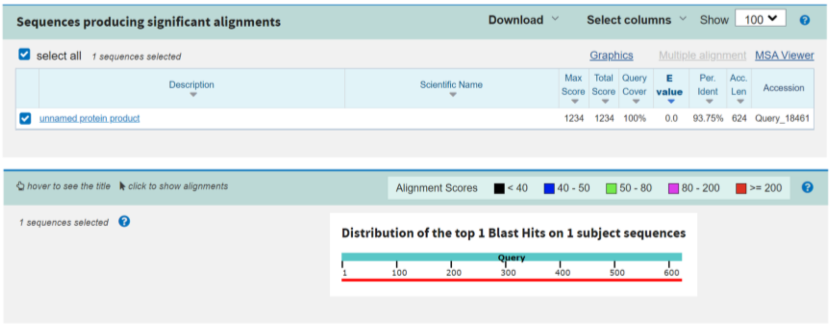

B.KEAP1 protein amino acid sequence DUI

```
Human    1 MQPDPRPSGA GACCRFLPLQ SQCPEGAGDA VMYASTECKA EVTPSQHGNR TFSYTLEDHT
Rat      1 MQPEPKPSGA  PRSSQFLPLW SKCPEGAGDA VMYASTECKA EVTPSQDGNR TFSYTLEDHT
Human   61 KQAFGIMNEL RLSQQLCDVT LQVKYQDAPA AQFMAHKVVL ASSSPVFKAM FTNGLREQGM
Rat     61 KQAFGIMNEL RLSQQLCDVT LQVKYE DIPA  AQFMAHKVVL ASSSPVFKAM FTNGLREQGM
Human  121 EVVSIEGIHP KVMERLIEFA YTASISMGEK CVLHVMNGAV MYQIDSVVRA CSDFLVQQLD
Rat    121 EVVSIEGIHP KVMERLIEFA YTASISV GEK CVLHVMNGAV MYQIDSVVRA CSDFLVQQLD
Human  181 PSNAIGIANF AEQIGCVELH QRAREYIYMH FGEVAKQEEF FNLSHCQLVT LISRDDLNVR
Rat    181 PSNAIGIANF AEQIGCTELH QRAREYIYMH FGEVAKQEEF FNLSHCQLAT LISRDDLNVR
Human  241 CESEVFHACI NWVKYDCEQR RFYVQALLRA VRCHSLTPNF LQMQLQKCEI LQSDSRCKDY
Rat    241 CESEVFHACI DWVKYDCPQR RFYVQALLRA VRCHALTPRF LQT  QLQKCEI LQADARCKDY
Human  301 LVKIFEELTL HKPTQVMPCR APKVGRLIYT AGGYFRQSLS YLEAYNPSDG TWLRLADLQV
Rat    301 LVQIFQELTL HKPTQAVPCR APKVGRLIYT AGGYFRQSLS YLEAYNPSNG SWLRLADLQV
Human  361 PRSGLAGCVV GGLLYAVGGR NNSPDGNTDS SALDCYNPMT NQWSPCAPMSVPRNRIGVGV
Rat    361 PRSGLAGCVV GGLLYAVGGR NNSPDGNTDS SALDCYNPMT NQWSPCASL  SVPRNRSGGGV
Human  421 IDGHIYAVGG SHGCIHHNSV ERYEPERDEW HLVAPMLTRR IGVGVAVLNR LLYAVGGFDG
Rat    421 IDGHIYAVGG SHGCIHHS SV ERYEPDRDEW HLVAPMLTRR IGVGVAVLNR LLYAVGGFDG
Human  481 TNRLNSAECY YPERNEWRMI TAMNTIRSGA GVCVLHNCIY AAGGYDGQDQ LNSVERYDVE
Rat    481 TNRLNSAECY YPERNEWRMI TPMNTIRSGA GVCVLHS CIY AAGGYDGQDQ LNSVERYDVE
Human  541 TETWTFVAPM KHRRSALGIT VHQGRIYVLG GYDGHTFLDS VECYDPDTDT WSEVTRMTSG
Rat    541 TETWTFVASM KHRRSALGIA VHQGRIYVLG GYDGHTFLDS VECYDPDTDT WSEVTRLTSG
Human  601   RSGVGVAVTM EPCRKQIDQQ NCTC
Rat    601 R9SGVGVAVTM EPCRKQIDQQ
```

Figure S2. Homology analysis of human KEAP1 protein and SD rat KEAP1 protein. **(A)** BLAST homology software analysis showed that the homology of amino acid sequence of KEAP1 protein in human and rat was up to 93.75%. **(B)** The comparison of amino acid sequences of human and rat KEAP1 protein showed that the same amino acid sequence could be found on the corresponding sites of rat KEAP1 protein at the docking sites between human KEAP1 protein and EG molecule. It showed that rat KEAP1 protein can also dock with EG at these sites.
